# Data-Adaptive Estimation for Double-Robust Methods in Population-Based Cancer Epidemiology: Risk Differences for Lung Cancer Mortality by Emergency Presentation

**DOI:** 10.1093/aje/kwx317

**Published:** 2018-01-30

**Authors:** Miguel Angel Luque-Fernandez, Aurélien Belot, Linda Valeri, Giovanni Cerulli, Camille Maringe, Bernard Rachet

**Affiliations:** 1 Faculty of Epidemiology and Population Health, Department of Non-Communicable Disease Epidemiology, Cancer Survival Group, London School of Hygiene and Tropical Medicine, London, United Kingdom; 2 Laboratory for Psychiatric Biostatistics, McLean Hospital, Belmont, Massachusetts; 3 Harvard Medical School, Harvard University, Boston, Massachusetts; 4 National Research Council of Italy, Research Institute on Sustainable Economic Growth, Rome, Italy

**Keywords:** cancer epidemiology, causality, machine learning, population-based data, statistics, targeted maximum likelihood estimation

## Abstract

In this paper, we propose a structural framework for population-based cancer epidemiology and evaluate the performance of double-robust estimators for a binary exposure in cancer mortality. We conduct numerical analyses to study the bias and efficiency of these estimators. Furthermore, we compare 2 different model selection strategies based on 1) Akaike’s Information Criterion and the Bayesian Information Criterion and 2) machine learning algorithms, and we illustrate double-robust estimators’ performance in a real-world setting. In simulations with correctly specified models and near-positivity violations, all but the naive estimators had relatively good performance. However, the augmented inverse-probability-of-treatment weighting estimator showed the largest relative bias. Under dual model misspecification and near-positivity violations, all double-robust estimators were biased. Nevertheless, the targeted maximum likelihood estimator showed the best bias-variance trade-off, more precise estimates, and appropriate 95% confidence interval coverage, supporting the use of the data-adaptive model selection strategies based on machine learning algorithms. We applied these methods to estimate adjusted 1-year mortality risk differences in 183,426 lung cancer patients diagnosed after admittance to an emergency department versus persons with a nonemergency cancer diagnosis in England (2006–2013). The adjusted mortality risk (for patients diagnosed with lung cancer after admittance to an emergency department) was 16% higher in men and 18% higher in women, suggesting the importance of interventions targeting early detection of lung cancer signs and symptoms.

Data from population-based cancer registries are critical for cancer control and policy (1–3). However, the scope of the information available from cancer registries is limited, pertaining only to cancer characteristics and basic sociodemographic factors (1, 2, 4). Recently, strategies for linkage of population-based data sets from different sources have been implemented. This has allowed for more advanced modeling scenarios regarding applications in cancer policy and control (5–10). For instance, comparative effectiveness approaches using medical records and linked population-based databases are used to evaluate the effectiveness of treatment or exposures concerning cancer mortality and survival (6–10). Nevertheless, evaluation of the effectiveness of treatments or exposures in a large population-based cancer study requires well-defined structural frameworks and modern statistical methods in order to overcome confounding (9).

The use of the Neyman-Rubin potential outcomes framework (11) allows researchers to make explicit the assumptions under which an observed association from observational studies can be interpreted causally. For a given factor to be considered causal, researchers must consider a set of additional assumptions (i.e., conditional exchangeability, positivity, and consistency) (12). Directed acyclic graphs (DAGs) help one to evaluate whether, under a given causal model, the counterfactual outcome is independent of the observed exposure given some set of covariates (conditional exchangeability) selected on the basis of subject matter knowledge (12–14).

The average treatment effect (ATE) or risk difference is a commonly used parameter of interest (12, 15, 16). Correct model specification is crucial to obtain unbiased estimates of the true ATE. Many estimators of the ATE (but not all) rely on parametric modeling assumptions, thereby introducing bias when the model is incorrect (15). Researchers have developed double-robust estimation procedures to reduce bias due to misspecification (17, 18). More recently, van der Laan and Rose (15, 19, 20) developed a targeted maximum likelihood estimation (TMLE) method using machine learning algorithms to minimize the risk of model misspecification. Simulation studies using TMLE in finite samples provide evidence of its double-robust properties and gains in performance when it is combined with machine learning algorithms (15, 21, 22).

However, there is no evidence evaluating the performance of TMLE compared with other double-robust methods in the setting of population-based cancer epidemiology. We sought to compare the performance of 3 different double-robust causal estimators of the ATE for cancer mortality in a simulated scenario with forced near-positivity violations (i.e., certain subgroups in the sample rarely or never receive treatment) and model misspecification. Furthermore, we studied the efficiency and bias of double-robust estimators and compared 2 different model selection strategies based on 1) a combination of Akaike’s Information Criterion (AIC) and the Bayesian Information Criterion (BIC) and 2) machine learning algorithms and TMLE. We illustrate these methods with real population-based data on lung cancer patients in England.

## METHODS

### Counterfactual framework

**Figure 1.**
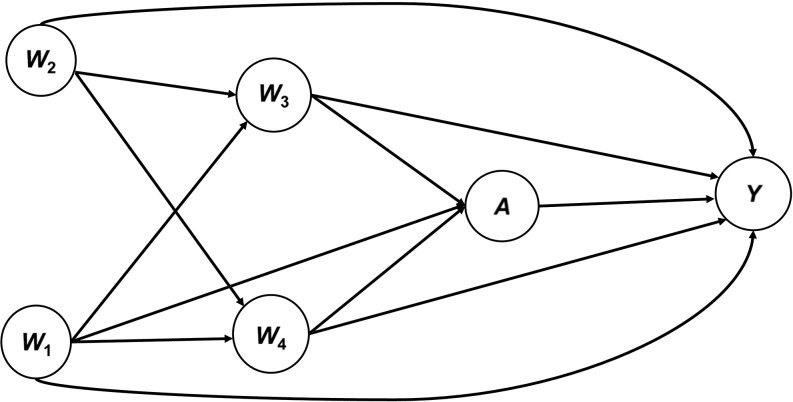
Directed acyclic graph for a proposed structural causal framework in population-based cancer research. Conditional exchangeability of the treatment effect or exposure (*A*) on 1-year cancer mortality (*Y*) is obtained through conditioning on a set of available covariates (*Y*_1_,*Y*_0_ ⊥ *A*|*W*). The minimum sufficient set, based on the backdoor criterion, is obtained through conditioning on only *W*_1_, *W*_3_, and *W*_4_. The average treatment effect for the structural framework is estimated as the average risk difference between the expected effect of the treatment conditional on *W* among treated persons (*E*(*Y*|*A* = 1; *W*)) and the expected effect of the treatment conditional on *W* among the untreated (*E*(*Y*|*A* = 0; *W*)). *W*_1_, socioeconomic status; *W*_2_, age; *W*_3_, cancer stage; *W*_4_, comorbidity.

On the basis of background knowledge, we used a DAG to depict our general counterfactual framework (Figure 1). We considered 1-year cancer mortality as a binary outcome *Y* and a generic binary exposure or treatment *A*, and we assumed that the following measured covariates were sufficient to ensure conditional exchangeability: patient’s socioeconomic status (*W*_1_), age (*W*_2_), cancer stage (*W*_3_), and comorbidity at diagnosis (*W*_4_) (Figure 1). Afterward, based on our DAG, we generated data to explore the effects of near-positivity violations and dual misspecification (outcome and treatment models). The set of covariates included in *W* is critical for cancer treatment decision-making (3, 23, 24). However, cancer stage and patient’s comorbidity at diagnosis play a crucial role in the choice of clinical treatment and have been cited as the most important explanatory factors for cancer mortality and survival (3, 23, 24). As depicted in our DAG, we highlighted the importance of patient’s cancer stage, socioeconomic status, and comorbidity as the minimum set of variables needed to assume conditional exchangeability based on the backdoor criterion. Our targeted parameter was the 1-year risk difference in cancer mortality for patients exposed to a generic exposure (*A*) versus nonexposed patients.

### Data-generation process and Monte Carlo simulations

We generated data based on the structural framework represented in Figure 1 by a DAG. The covariates (*W*) were drawn using a set of random uniform and binomial variables. The propensity score for the binary exposure (*A*) and the outcome variable (*Y*) were derived from a binomial logit model that included the interaction between age (*W*_2_) and comorbidity (*W*_4_) for the generation of *Y*.

Afterward, we drew 1,000 replications from the data-generation process with sample sizes of 1,000 and 10,000. In each replication, we estimated the binary ATE and recorded the point estimates and standard errors based on the influence curve in order to calculate the ATE standard deviations, bias, 95% confidence interval coverage, and root mean squared error (25).

### Model estimation scenarios and performance evaluation

**Figure 2.**
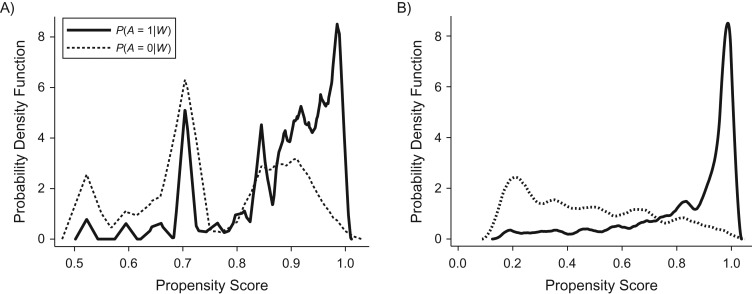
Overlap of the propensity scores for correctly specified (first scenario (A)) and misspecified (second scenario (B)) models for the probabilities of treatment status *P*(*A* = 1|*W*) and *P*(*A* = 0|*W*) in 1 random sample from 1,000 Monte Carlo simulations.

We set 2 different modeling scenarios aiming to assess the performance of double-robust estimators of the ATE using 1) correctly specified models for the treatment and the outcome and 2) misspecified models for both treatment and outcome. Correctly specified models for the treatment and outcome included socioeconomic status (*W*_1_), age (*W*_2_), cancer stage (*W*_3_), and comorbidity (*W*_4_) as covariates. Model misspecification for the treatment and the outcome was forced omitting the interaction between comorbidity (*W*_4_) and age (*W*_2_). Data-adaptive approaches were used to estimate the treatment and outcome for misspecified models (Web Appendix 1, available at https://academic.oup.com/aje, describes in more detail the model specifications for the data generation). For both scenarios, we included near-positivity violations that forced some values of the propensity score distribution close to zero. Near-positivity violations were evaluated visually based on the summary of the propensity score distribution. Figure 2 illustrates the overlap of the distribution of the potential outcomes for one simulated sample in the first scenario (Figure 2A) and the second scenario (Figure 2B).

In the first scenario, which uses correctly specified models, we evaluated the performance of a classical multivariate regression adjustment with treatment (*A*) and covariates (*W*_1_–*W*_4_) as predictors of the outcome (*Y*), namely the naive approach, and of 3 different double-robust estimators of the ATE: 1) inverse-probability-of-treatment-weighted regression adjustment (IPTW-RA) (26); 2) augmented inverse-probability-of-treatment weighting (AIPTW) (17, 27, 28); and 3) TMLE (15, 29). IPTW-RA is a regression model weighted by the inverse probability of treatment, whereas AIPTW is a 2-step procedure with 2 estimating equations for the treatment and mean outcome, respectively (27).

For the second scenario, using misspecified models, we evaluated 2 different data-adaptive model selection strategies in combination with the above-described double-robust estimators. Models for the treatment and outcome included the above-described covariates for the first scenario but omitted the interaction between comorbidity and age used to generate the data in the second scenario. (Web Appendix 1 describes model specifications for the data generation in more detail.) As data-adaptive strategies, we used AIC-BIC approaches for the IPTW-RA and AIPTW estimators and ensemble learning for the TMLE estimator. For the IPTW-RA, we used the AIC-BIC-based approach implemented in the STATA user-written command “bfit” (best fit) (30). The “bfit” algorithm sorts a set of fitted candidate regression models using the AIC and BIC and displays a table showing the ranking of the models. Each linear predictor of the candidate models is defined as a linear combination of functional forms of the variables. The smallest of the candidate models includes only 1 variable. The largest of the candidate models includes all of the variables in a fully interacted polynomial of the order prespecified by the user. We set the order to “2” for comparative purposes with TMLE. For simulations and analysis of the IPTW-RA and AIPTW estimators, we used STATA, version 14.1 (StataCorp LP, College Station, Texas) and the “teffects ipwra” and “teffects aipw” commands (26).

The TMLE estimator has not been implemented in STATA statistical software yet, so we used the package “tmle” (version 1.2.0-4) (29) from R, version 3.0.2 (R Foundation for Statistical Computing, Vienna, Austria). The implementation of TMLE in R loads the SuperLearner package. SuperLearner uses V-fold (10-fold by default) cross-validation to assess the performance of the prediction of the outcome and the propensity score models as weighted averages (ensemble learning) of a set of machine learning algorithms (29, 31). We used the default specifications of the “tmle” package, which included the following machine learning algorithms: 1) stepwise forward and backward selection; 2) generalized linear modeling (“glm”) with the covariates (*W*) and the treatment (*A*) as main terms; and 3) a “glm” variant that included second-order polynomials and 2-by-2 interactions of the main terms included in the model. In Web Appendix 2, we provide a basic implementation of the TMLE algorithm in both STATA and R statistical software, as well as the link to a testing version of TMLE implemented in STATA.

### Monte Carlo simulation results

#### First scenario: correctly specified models and near-positivity violation

The true risk difference of the ATE estimate from the 1,000 simulation repetitions was −18%. The naive approach showed a biased estimate of the ATE with overestimation of the treatment effect by 23% (relative bias). All double-robust estimators were nearly unbiased, showing a smaller root mean squared error with increasing sample size, but the TMLE presented higher precision (based on the difference in variances between estimators), the smallest root mean squared error, and the best coverage (95%) (Table 1, first scenario: correctly specified models).

**Table 1.** Results From 10,000 Monte Carlo Simulations of the Average Treatment Effect for Correctly Specified Models (First Scenario) and Misspecified Models Using Adaptive Approaches (Second Scenario) for Different Double-Robust Estimators of 1-Year Lung Cancer Mortality, England, 2006–2013

Simulated Scenario	ATE^a^ (SD)		Absolute Bias		Relative Bias, %		RMSE		95% CI Coverage, %	
	*n* = 1,000	*n* = 10,000	*n* = 1,000	*n* = 10,000	*n* = 1,000	*n* = 10,000	*n* = 1,000	*n* = 10,000	*n* = 1,000	*n* = 10,000
First scenario^b^										
True ATE	−0.1813									
Naive	−0.2234 (0.049)	−0.2218 (0.012)	0.0421	0.0405	23.2	22.3	0.0575	0.0423	77	89
AIPTW	−0.1843 (0.053)	−0.1848 (0.018)	0.0030	0.0035	1.6	1.9	0.0534	0.0180	93	94
IPTW-RA	−0.1831 (0.050)	−0.1838 (0.017)	0.0018	0.0025	1.0	1.4	0.0500	0.0174	91	95
TMLE^c^	−0.1832 (0.048)	−0.1821 (0.016)	0.0019	0.0008	1.0	0.4	0.0482	0.0158	95	95
Second scenario^d^										
True ATE	−0.1172									
Naive	−0.0127 (0.103)	−0.0121 (0.033)	0.1045	0.1051	89.2	89.7	0.1470	0.1100	0	0
BF^e^ AIPTW	−0.1155 (0.093)	−0.0920 (0.073)	0.0017	0.0252	1.5	11.7	0.0928	0.0773	65	65
BF^e^ IPTW-RA	−0.1268 (0.043)	−0.1192 (0.031)	0.0096	0.0020	8.2	1.7	0.0442	0.0305	52	73
TMLE^c^	−0.1181 (0.028)	−0.1177 (0.011)	0.0009	0.0005	0.8	0.4	0.0281	0.0107	93	95

Abbreviations: AIPTW, augmented inverse-probability-of-treatment weighting; ATE, average treatment effect; BF, best fit; CI, confidence interval; IPTW-RA, inverse-probability-of-treatment-weighted regression adjustment; RMSE, root mean squared error; SD, standard deviation; TMLE, targeted maximum likelihood estimation.

^a^ ATE across 1,000 simulated data sets.

^b^ First scenario: correctly specified models and near-positivity violation.

^c^ TMLE calling basic SuperLearner (SL) libraries: SL.Step, SL.glm, and SL.glm.interaction.

^d^ Second scenario: misspecification, near-positivity violation, and adaptive model selection.

^e^ Best fit based on Akaike’s Information Criterion and the Bayesian Information Criterion.

#### Second scenario: misspecification, near-positivity violation, and adaptive model selection

The true risk difference of the ATE from the 1,000 simulation repetitions was −12%. The naive approach was heavily biased, showing the highest root mean squared error with underestimation of the treatment effect by approximately 90% (Table 1, second scenario: adaptive estimation approach). The model selection strategy based on AIC-BIC did not show either bias reduction or coverage improvement. The double-robust TMLE estimator presented the best performance with more precise estimates (1% bias for a sample size of 1,000 patients and less than 0.5% for a sample size of 10,000 patients) and the highest coverage. By contrast, the relative bias increased with increasing sample size for the AIPTW estimator using the AIC-BIC approach. The relative bias ranged from 1.5% (*n* = 1,000) to 11.7% (*n* = 10,000) (Table 1, second scenario).

## ILLUSTRATION

Under the structural framework (see DAG in Figure 1) described above for population-based cancer epidemiology, we estimated 1-year adjusted mortality risk differences for cancer diagnosed after admittance to a hospital emergency department versus receiving a nonemergency cancer diagnosis. The high proportion of lung cancer diagnosed after admittance to an emergency department in England (emergency presentation) has been hypothesized to be mainly due to multiple steps that patients undergo between identification of the first symptoms and final diagnosis by the health-care system.

In addition to age and socioeconomic status, we included comorbidity and cancer stage as confounders. Evidence shows that the presence of patient comorbidity increases the odds of being diagnosed with distant metastases (advanced cancer stage), and it does not lead to an earlier cancer diagnosis (32). Socioeconomic status was measured using quintiles of the income domain of the Index of Multiple Deprivation in England (33); the presence of comorbid conditions was based on the Charlson Comorbidity Index (34); and stage was based on the tumor-node-metastasis classification of malignant tumors (35). In England, a cancer diagnosis after emergency presentation correlates closely with poor 1-year survival. However, the strength of the evidence comes from observational data and is weak, owing to confounding (36).

It is of public health interest to estimate the 1-year adjusted mortality risk differences for cancer diagnosed after an emergency presentation, given the potential impact of a preventive intervention aiming to improve earlier cancer diagnosis. Quantifying the sex-specific adjusted risk differences in 1-year mortality for lung cancer patients will reinforce the current evidence and help to promote the policy actions required for improving early cancer diagnoses.

To illustrate estimation of the adjusted risk differences for 1-year mortality, we extracted data from the United Kingdom’s National Cancer Data Repository for 183,426 incident cases of lung cancer (102,535 men and 80,891 women) diagnosed in England between 2006 and 2013. All patients had a minimum potential follow-up period of 1 year, since vital status was not assessed until December 31, 2014. Data were restricted to cases with complete information on sex, age at diagnosis, comorbidity, cancer stage, socioeconomic deprivation, and type of cancer diagnosis. The strategy for assessment of a cancer diagnosis after an emergency department presentation has been previously described (37). Overall, more than 80% of the patients who died within 1 year after a cancer diagnosis had been diagnosed after an emergency department presentation, and only 96 (representing 0.05%) were lost to follow-up before 1 year (Web Table 1). The average age at diagnosis was 72 years in men and 73 years in women. One-year mortality after diagnosis showed a balanced distribution across the different age and socioeconomic groups and across quartiles of the Charlson Comorbidity Index (34). However, persons with stages IV and III cancer had 4- and 3-fold higher probabilities of 1-year mortality, respectively, than those with stage I cancer (Table 2).

**Table 2.** One-Year Mortality Among Lung Cancer Patients (Incident Cases; *n* = 183,426 (102,535 Males and 80,891 Females)), by Cancer Stage, Comorbidity, Socioeconomic Status, and Age at Cancer Diagnosis, After Admittance to an Emergency Department Versus Nonemergency Diagnosis, England, 2006–2013

Variable	Mortality 1 Year After Diagnosis, %	
	Women	Men
ER presentation		
No	53.4	59.9
Yes	83.7	86.4
Cancer stage		
I	18.1	24.2
II	35.1	37.6
III	58.6	62.4
IV	82.2	85.8
Quartile of CCI		
1 (lowest)	62.8	67.6
2	64.1	68.3
3	67.2	71.4
4 (highest)	72.4	75.5
Quintile of SES		
1 (lowest)	62.6	66.7
2	63.3	68.1
3	64	69.5
4	64.2	69.6
5 (highest)	64.1	68.2
Age at diagnosis, years^a^	73.0 (10.8)	72.6 (10.3)

Abbreviations: CCI, Charlson Comorbidity Index; ER, emergency room; SES, socioeconomic status.

^a^ Values are presented as mean (standard deviation).

**Figure 3.**
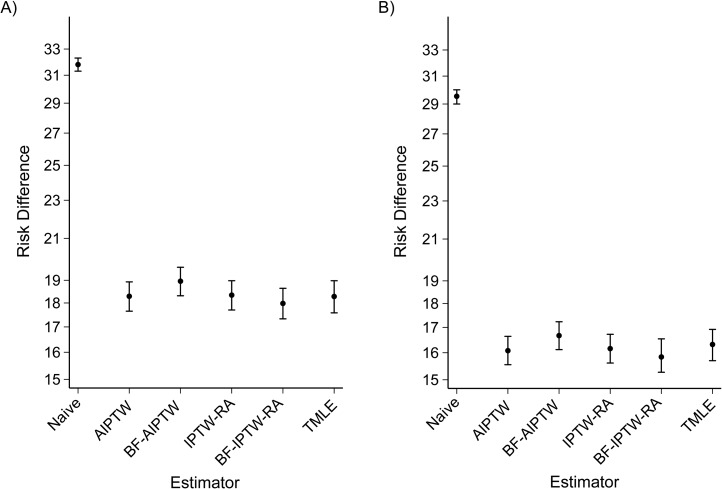
Sex-specific adjusted risk difference for 1-year lung cancer mortality according to different double-robust estimators among 183,426 lung cancer patients diagnosed after admittance to an emergency department versus persons with a nonemergency cancer diagnosis, England, 2006–2013. A) women; B) men. Bars, 95% confidence intervals. AIPTW, augmented inverse-probability-of-treatment weighting; BF-AIPTW, best-fit augmented inverse-probability-of-treatment weighting (data-adaptive estimation based on Akaike’s Information Criterion (AIC) and the Bayesian Information Criterion (BIC)); BF-IPTW-RA, best-fit inverse-probability-of-treatment-weighted regression adjustment (data-adaptive estimation based on AIC-BIC); IPTW-RA, inverse-probability-of-treatment-weighted regression adjustment; TMLE, targeted maximum likelihood estimation (data-adaptive estimation based on ensemble learning and *k*-fold cross-validation).

To estimate the adjusted mortality risk difference, we used the same approaches and commands as those used for the simulation study. We provide commented code for the illustration in Web Appendix 2. Overall, based on double-robust estimators, we determined that the adjusted risk of 1-year mortality between cancer diagnosed after admittance to an emergency department versus a nonemergency diagnosis was 16% higher in men and 18% higher in women than it was after nonemergency diagnosis. However, the naive approach showed the largest risk difference, with 29% and 32% adjusted risk differences for women and men, respectively (Figures 3A (women) and 3B (men)).

We also used the observed covariates from the illustration to run 100 Monte Carlo simulations to estimate the adjusted mortality risk difference for 1-year cancer mortality after admittance to an emergency department. Using the information on baseline covariates from the observed data, we simulated only the outcome and treatment models. To evaluate the performance of the different estimators under strong near-positivity violations, we forced some values of the propensity scores close to zero (Web Figure 1). However, the estimation models for the treatment and outcome were correctly specified during simulations to include the interaction between age and comorbidity (we provide the model specifications and the variables included for the simulations in Web Appendix 1). The propensity score distributions among the exposed and unexposed overlapped considerably in the real-world setting (Web Figure 1A), while the overlap in the simulated scenario was poor given the strong near-positivity violation (Web Figure 1B). Table 3 presents the results of the simulations, which validate the previous results with similar findings, but with a larger sample size and fixed covariates coming from a real-world scenario, thus reproducing reality much better. TMLE presented the best precision and coverage and outperformed all other double-robust estimators. By contrast, AIPTW showed high sensitivity to the violation of the positivity assumption, with a relative bias of 8% (Table 3).

**Table 3.** Results of a Monte Carlo Simulation of Risk Differences in 1-Year Mortality Among Lung Cancer Patients (Incident Cases; *n* = 183,426) Diagnosed After Admittance to an Emergency Department, England, 2006–2013

Estimator	ATE^a^ (SD)	Absolute Bias	Relative Bias, %	RMSE	95% CI Coverage, %
True ATE	0.1621				
AIPTW	0.1493 (0.010)	0.0128	7.9	0.0165	79
IPTW-RA	0.1587 (0.006)	0.0034	2.1	0.0072	92
TMLE^b^	0.1620 (0.003)	0.0001	0.1	0.0034	92

Abbreviations: AIPTW, augmented inverse-probability-of-treatment weighting; ATE, average treatment effect; CI, confidence interval; IPTW-RA, inverse-probability-of-treatment-weighted regression adjustment; RMSE, root mean squared error; SD, standard deviation; TMLE, targeted maximum likelihood estimation.

^a^ ATE across 1,000 simulated data sets.

^b^ TMLE calling basic SuperLearner (SL) libraries: SL.Step, SL.glm, and SL.glm.interaction.

## DISCUSSION

Given the increasing availability of a different range and variety of data in population-based cancer epidemiology, the proposed structural framework (Figure 1) constitutes a basis for further development of comparative effectiveness research in population-based cancer epidemiology. Developed for a binary treatment and outcome, the framework can be easily extended to handle time-to-event outcomes and might be adapted to specific comparative effectiveness scenarios. For instance, we considered cancer patients’ comorbidity and stage as confounders, but this might not be the case with other comparative effectiveness research questions. We recently published an article in which we argued that multivariate adjustment for cancer-related comorbid conditions (those with an onset date close before or after the date of cancer diagnosis) to evaluate the effectiveness of cancer treatment might be inappropriate, as it could induce collider stratification bias (38).

We also applied the proposed structural framework (Figure 1) to a real-world data scenario and highlighted the critical importance of considering cancer stage and patient’s comorbidity in the structural framework to satisfy the conditional exchangeability assumption in population-based cancer epidemiology. Conventional methods control for confounding by assuming that the effect measure of the exposure of interest is constant across all levels of the covariates included in the model (39). We provided evidence of highly imprecise estimates of ATE in the classical naive regression method, underestimating the effect of the treatment, particularly for the misspecified model in the simulation setting.

Model misspecification with parametric modeling is always a concern in epidemiologic research. ATE estimators based on the propensity score or regression adjustment are unbiased only if estimation models are correctly specified (17, 27, 40). Double-robust estimation combines these two approaches so that only 1 of the 2 models needs to be correctly specified to obtain an unbiased estimate of the ATE (17, 27, 40). Previous simulation studies have shown that double-robust methods, including TMLE, consistently provide almost unbiased estimates when either the propensity score or the outcome model is misspecified but the other is correct (41–43). However, more evidence is needed to evaluate TMLE statistical properties under different modeling scenarios.

TMLE is a general algorithm that can utilize the g-formula (44) as a generalization of standardization, defining the parameters of interest semiparametrically as a function of the data-generating distribution. TMLE evaluates the target parameter (ATE) by using a double-robust semiparametric substitution estimation based on machine learning algorithms to avoid misspecification and reduce bias (22).

Our results showed that when the models were correctly specified, standardization implemented through IPTW-RA, AIPTW, and TMLE provided nearly unbiased estimates of the ATE, despite near-positivity violations. TMLE, however, was the most efficient estimator. Nevertheless, dual misspecification is the likely scenario in population-based cancer epidemiology; thus, attempting to obtain the best possible estimates is paramount for policy recommendations. Under dual misspecification and near-positivity violations, both in simulations and in a real-life illustration, AIPTW showed poorer performance than IPTW-RA and TMLE, illustrating the instability of AIPTW to estimate values of the propensity score close to zero (near-positivity violations) as previously reported by Kang and Schafer (27). However, basic machine learning algorithms and ensemble learning techniques implemented in the “tmle” and SuperLearner R packages avoid misspecification of the models (for either the treatment or the outcome) used to estimate the ATE.

To the best of our knowledge, the performance of double-robust methods using different model selection strategies has not been evaluated in the context of adverse estimation situations with near-violations of the positivity assumption and misspecified models. Based on a simulated scenario, we compared the STATA user-written program “bfit” (30) with machine and ensemble learning algorithms implemented in the R package “tmle” based on SuperLearner (29, 45). TMLE outperformed model selection strategies based on AIC-BIC for the IPTW-RA and AIPTW estimators. By default, TMLE implementation in R sets a bounded distribution of the propensity score to 0.025 and 0.975, and the adaptive estimation respects the limits of the possible range of the targeted parameter, but AIPTW does not. So AIPTW could, for instance, produce estimates that are outside the range of the targeted parameter. Moreover, the default AIPTW implementation in STATA will not converge for very small values of the propensity score with a tolerance set by default to 10^−5^. We had to increase the tolerance of the weights for the propensity score to 10^−8^ when using the AIC-BIC adaptive approach (STATA “bfit”) for the AIPTW estimator, given convergence problems associated with the near-positivity violations. The relative bias using an adaptive approach based on AIC-BIC for AIPTW estimation under difficult scenarios increases with a larger sample size (from 1,000 to 10,000 in our simulation setting). Hence, using AIC-BIC for the AIPTW estimator might not be a good option when there is a strong suspicion of model misspecification and near-violation of the positivity assumption. Further evidence is needed to evaluate our findings.

However, the performance of AIPTW is similar to that of IPTW-RA and TMLE under certain scenarios (correct specification and without near-positivity violations). TMLE is computationally demanding, manifesting in slow run times for large cancer population data (e.g., using a computer with 4 cores and 16 GB of memory, the R package “tmle” took 5.4 minutes to estimate the ATE for 10,000 patients using more advanced machine learning algorithms such as generalized additive models, random forests, and boosting).

Under an adverse estimation scenario, with near-positivity violations and dual misspecification, the TMLE estimator of the ATE for a binary treatment and outcome performs better than other double-robust estimators. Its reductions in bias and gains in efficiency support the use of TMLE for a binary treatment and outcome in population-based cancer epidemiology research. Results from the illustration provide quantitative evidence of an increased 1-year mortality risk in patients diagnosed with lung cancer after visiting a hospital emergency department. This finding should boost calls for policy interventions such as the implementation of multidisciplinary diagnosis centers to improve early cancer diagnosis and management.

## Supplementary Material

Web Material

## Abbreviations

AIC: Akaike’s Information Criterion
AIPTW: augmented inverse-probability-of-treatment weighting
ATE: average treatment effect
BIC: Bayesian Information Criterion
DAG: directed acyclic graph
IPTW-RA: inverse-probability-of-treatment-weighted regression adjustment
TMLE: targeted maximum likelihood estimation
