# Supplementary material for: Data-Adaptive Estimation for Double-Robust Methods in Population-Based Cancer Epidemiology: Risk Differences for Lung Cancer Mortality by Emergency Presentation
Source: Am J Epidemiol. 2018 Jan 30;187(4):871–8. doi: 10.1093/aje/kwx317 (PMC5888939; doi:10.1093/aje/kwx317)

WEB APPENDIX 1

Data-Generation with Stata

On the basis of the structural causal model from Figure 1, data were drawn independently from the following distributions (**w1**=Socioeconomic status, **w2**=Age, **w3**=Cancer stage, **w4**=Comorbidities):

```
gen w1  =  round(runiform(1, 5)) //Quintiles of Socioeconomic status
gen w2  =  rbinomial(1, 0.45) //Binary: probability age >65 = 0.45
gen w3  =  round(runiform(0, 1) + 0.75*(w2) + 0.8*(w1)) //Stage
recode w3 (5/6=1) //Stage (TNM): categorical four levels
gen w4  = round(runiform(0, 1) + 0.75*(w2) + 0.2*(w1)) //Comorbidities: categorical four levels
```

<u>Scenario 1</u>

Data for the binary treatment model

```
gen A = (rbinomial(1,invlogit(-1 -  0.15*(w4) + 1.5*(w2) + 0.75*(w3) + 0.25*(w1))))
```

Data for the binary outcome model including the interaction between age and comorbidities

```
gen Y = (rbinomial(1,invlogit(-3 + A + 0.25*(w4) + 0.75*(w3) + 0.8*(w2)*(w4) + 0.05*(w1))))
```

<u>Scenario 2</u>

```
gen cosw2 = cos(2*w2)
gen sinw3  = sin(4*w3)
```

Data for the binary treatment model including the interaction between age and comorbidities

```
gen A = (rbinomial(1,invlogit(-1 - 0.3*(w4)  - 0.1*(w3) + 2*(cosw2)*w4 + 1.1*(w1))))
```

Data for the binary outcome model including the interaction between age and comorbidities

```
gen Y = (rbinomial(1,invlogit(-5 + A  + 0.1*(cosw2) + 0.1*(sinw3) + 1.3*(w4) + 0.1*(w3) +
2.1*(w2)*(w4) + 0.5*(w1))))
```

<u>Simulated scenario based on the illustration</u>

Using the data from the emergency presentations illustration, in addition to the variables age, stage and comorbidities (**w1**=Socioeconomic status, **w2**=Age, **w3**=Cancer stage, **w4**=Comorbidities) we created the following covariates:

```
gen cosw4=cos(_pi*w4)
gen sinw3=sin(w3)
recode w2 0/74.99=1 75.00/105=2
gen w2sq =w2^2
```

Model for the binary treatment including the interaction between age and comorbidities

```
gen A = (rbinomial(1,invlogit(1 + 2*(cosw4) + 2.2*(sinw3) - 0.01*(w2)*(w4))))
```

Model for the binary outcome

```
gen Y = (rbinomial(1,invlogit(-2 + A - 0.5*(cosw4) + 1.5*(sinw3) + 0.65*(w2sq) + 0.3*(w1))))
```

WEB APPENDIX 2

1. TMLE algorithm in R and Stata software implementation

A) R code for the TMLE estimation of the ATE parameter

```
#---------------------------------------------------------------
# Generate Data - function to generate the data
# input: number of draws
# output: ObsData + counterfactuals
#---------------------------------------------------------------

generateData<- function(n){
  w1 <- rbinom(n, size=1, prob=0.5)
  w2 <- rbinom(n, size=1, prob=0.65)
  w3 <- runif(n, min=0, max=1)
  A   <- rbinom(n, size=1, prob= plogis(-0.4 + 0.2*w1 + 0.5*w2))
  Y   <- rbinom(n, size=1, prob= plogis(-1 + A -0.1*w1 + 0.3*w2 + 0.25*w3))

  # counterfactual
  Y.1<- rbinom(n, size=1, prob= plogis(-1 + 1 -0.1*w1 + 0.3*w2 + 0.25*w3))
  Y.0<- rbinom(n, size=1, prob= plogis(-1 + 0 -0.1*w1 + 0.3*w2 + 0.25*w3))

  # return data.frame
  data.frame(w1, w2, w3, A, Y, Y.1, Y.0)
}

#---------------------------------------------------------------------------
# Targeted Maximum Likelihood to estimate the ATE (Psi)
#---------------------------------------------------------------------------
set.seed(777)
ObsData<- generateData(n=10000)
True_Psi <- mean(ObsData$Y.1-ObsData$Y.0);True_Psi

ObsData<- subset(ObsData, select=c(w1,w2,w3,A,Y))
Y   <- ObsData$Y
A   <- ObsData$A
w1 <- ObsData$w1
w2 <- ObsData$w2
w3 <- ObsData$w3

#Step 1: prediction model for the outcome

m <- glm(Y ~ A + w1 + w3, family = binomial, data=ObsData)

Q <- cbind(QAW = predict(m),
           Q1W = predict(m, newdata = data.frame(A = 1, w1, w3)),
           Q0W = predict(m, newdata = data.frame(A = 0, w1, w3)))

#Step 2: prediction model for the treatment

g <- glm(A ~ w1 + w2, family = binomial)
g1w = predict(g, type ="response")

#Step 3: estimating the substitution parameter (epsilon)

h <- cbind(A / g1w - (1 - A) / (1 - g1w), 1 / g1w, - 1 / (1 - g1w))
epsilon <- coef(glm(Y ~ -1 + h[,1] + offset(Q[,"QAW"]), family = binomial))

#Step 4: updating the first estimate of the E0(Y|A, W)

Qstar <- plogis(Q + epsilon*h)

#Step 5: estimating the updated ATE (ψ1))

Psi <- mean(Qstar[,"Q1W"] - Qstar[,"Q0W"]);Psi
```

B) Syntax for the ATE using the R package "tmle"

```
#-------------------------------------------------------
# R package tmle to estimate the ATE (Psi)
#-------------------------------------------------------

library("tmle")
w <- subset(ObsData, select=c(w1,w2,w3))
tmle <- tmle(Y=ObsData$Y, A=ObsData$A, W=w, family="binomial")
summary(tmle)
names(tmle)
tmle$epsilon
tmle$estimate$ATE
```

C) Stata code for the TMLE estimation of the ATE parameter

```
import delimited ObsData.csv, clear
gen true= y1- y0
mean true
```

***Step 1: prediction model for the outcome**

```
glm y a w1 w3, fam(binomial)
predict double QAW, xb
gen aa=a
replace a = 0
predict double Q0W, xb
replace a = 1
predict double Q1W, xb
replace a = aa
drop aa
```

***Step 2: prediction model for the treatment**

```
glm a w1 w2, fam(binomial)
predict g
gen h = a / g  - (1 - a) / (1 - g)
gen double h1 = 1 / g
gen double h0 = - 1 / (1 - g)
```

***Step 3: estimating the substitution parameter (epsilon)**

```
glm y h, fam(binomial) offset(QAW) noconstant
mat a= e(b)
gen epsilon = a[1,1]
```

***Step 4: updating the first estimate of the E_0(Y|A, W)**

```
gen qstar = exp(h*epsilon + QAW) / (1 + exp(h*epsilon + QAW))

gen double qstar0 = exp(h0*epsilon + Q0W) / (1 + exp(h0*epsilon + Q0W))
gen double qstar1 = exp(h1*epsilon + Q1W) / (1 + exp(h1*epsilon + Q1W))
```

***Step 5: Estimating the updated ATE (ψ_1)**

```
gen psi = (qstar1 - qstar0)
mean psi
```

2. Code used for the illustration

A) STATA bfit, teffect ipwra, and teffect aipvw

Codebook variables

Time: time since diagnosis
EP: emergency presentation, binary indicator, 1 EP, 0 Non-EP.
CCI_4fact: quartiles of the Charlson's index score.
Dep: quintiles of deprivation
Agediag: age in years at diagnosis
Sex: 1 female, 2 male
Tnm: Stage, factor of 4 levels.
Dead_1y: One-year mortality indicator, 1 death, 0 alive.

```
*Naive approach
stset time_1y, fail(dead_1y)
stcox EP CCI_4fact dep tnm agediag
stcox EP CCI_4fact dep tnm agediag if sex == 1
stcox EP CCI_4fact dep tnm agediag if sex == 2

*Naive approach risk differences
stmh EP tnm CCI_4fact dep agediag, by(sex)
cs EP dead_1y if sex==1
cs EP dead_1y if sex==2

*Naive approach: risk ratios
glm dead_1y EP CCI_4fact dep tnm agediag if sex == 1, exp(time_1y) fam(poiss) link(log) eform
glm dead_1y EP CCI_4fact dep tnm agediag if sex == 2, exp(time_1y) fam(poiss) link(log) eform

*Best fit estimation of the ATE
clear
use "H:\Desktop\Lung_EP_data_2006-13.dta"

bfit logit EP tnm CCI_4fact dep agediag, corder(2)
local vlist "`r(bvlist)'"
display "vlist contains `vlist'"
estimates replay `r(bmodel)'

forvalues i = 1/2{
teffects ipwra (dead_1y tnm dep agediag CCI_4fact) (EP `vlist') if sex==`i'
nlcom 100*_b[ATE:r1vs0.EP]/_b[POmean:0.EP]
}

forvalues i = 1/2{
teffects aipw (dead_1y tnm dep agediag CCI_4fact) (EP `vlist') if sex==`i'
nlcom 100*_b[ATE:r1vs0.EP]/_b[POmean:0.EP]
}

*ATE estimation without bfit
forvalues i=1/2{
teffects ipwra (dead_1y tnm dep agediag CCI_4fact) (EP tnm dep agediag CCI_4fact) if sex==`i'
nlcom 100*_b[ATE:r1vs0.EP]/_b[POmean:0.EP]
}

forvalues i=1/2{
teffects aipw (dead_1y tnm dep agediag CCI_4fact) (EP tnm dep agediag CCI_4fact) if sex==`i'
nlcom 100*_b[ATE:r1vs0.EP]/_b[POmean:0.EP]
}
```

B) R TMLE

Variable codebook

A: emergency presentation, binary indicator, 1 EP, 0 Non-EP.
Y: one-year mortality indicator, 1 death, 0 alive.
W1: quintiles of deprivation
W2: age in years at diagnosis
W3: stage, factor of 4 levels.
W4: quartiles of the Charlson's index score.
Sex: "f" female, "m" male.

```
#Data
data <- TMLE_LUNG

#ATE MALES
datam <- data[data$sex == "m",]
w <- subset(datam, select = c(w1,w2,w3,w4) )
A <- datam$A
Y <- datam$Y

#Estimation:
tmle <- tmle(Y = Y, A = A, W = w, family = "binomial",
        Q.SL.library = c("SL.glm", "SL.step", "SL.glm.interaction"),
        g.SL.library  = c("SL.glm", "SL.step", "SL.glm.interaction"))
summary(tmle)

#ATE FEMALES
dataf <- data[data$sex == "f",]
w <- subset(dataf, select = c(w1,w2,w3,w4) )
A <- dataf$A
Y <- dataf$Y
#Estimation:
tmle <- tmle(Y = Y, A = A, W = w, family = "binomial",
        Q.SL.library = c("SL.glm", "SL.step", "SL.glm.interaction"),
        g.SL.library = c("SL.glm", "SL.step", "SL.glm.interaction"))
summary(tmle)
```

3. Link to testing version of TMLE implemented in STATA

https://github.com/migariane/meltmle

https://github.com/migariane/weltmle

Web Table 1. Description of lung cancer incident cases diagnosed in England between 2006 and 2013 (n = 183,426)

Covariates in Analysis		***N* (%)**
Deceased*		
	No	61,571 (33.6)
	Yes	121,855 (66.4)
Emergency presentation		
	No	122,219 (66.6)
	Yes	61,207 (33.4)
Sex		
	Male	102,535 (55.9)
	Female	80.891 (44.1)
Cancer stage at diagnosis		
	I	24,066 (13.1)
	II	13,570 (7.4)
	III	46,908 (25.6)
	IV	98,882 (53.9)
Quartiles of cancer comorbidity index		
	Q1	127,776 (69.7)
	Q2	28,445 (15.5)
	Q3	20,562 (11.2)
	Q4	6,643 (3.6)
Quintiles of socioeconomic status		
	Q1	25,277 (13.8)
	Q2	31,432 (17.1)
	Q3	36,298 (19.8)
	Q4	43,349 (23.6)
	Q5	47,120 (25.7)
Age at diagnosis, years		
	<75	108,750 (59.3)
	≥75	74,676 (40.7)

* 96 (0.05%) cancer patients were lost to follow-up before 1 year.

Web Figure 1. Overlap of the propensity score for the real observed data (A) and simulated data (B).

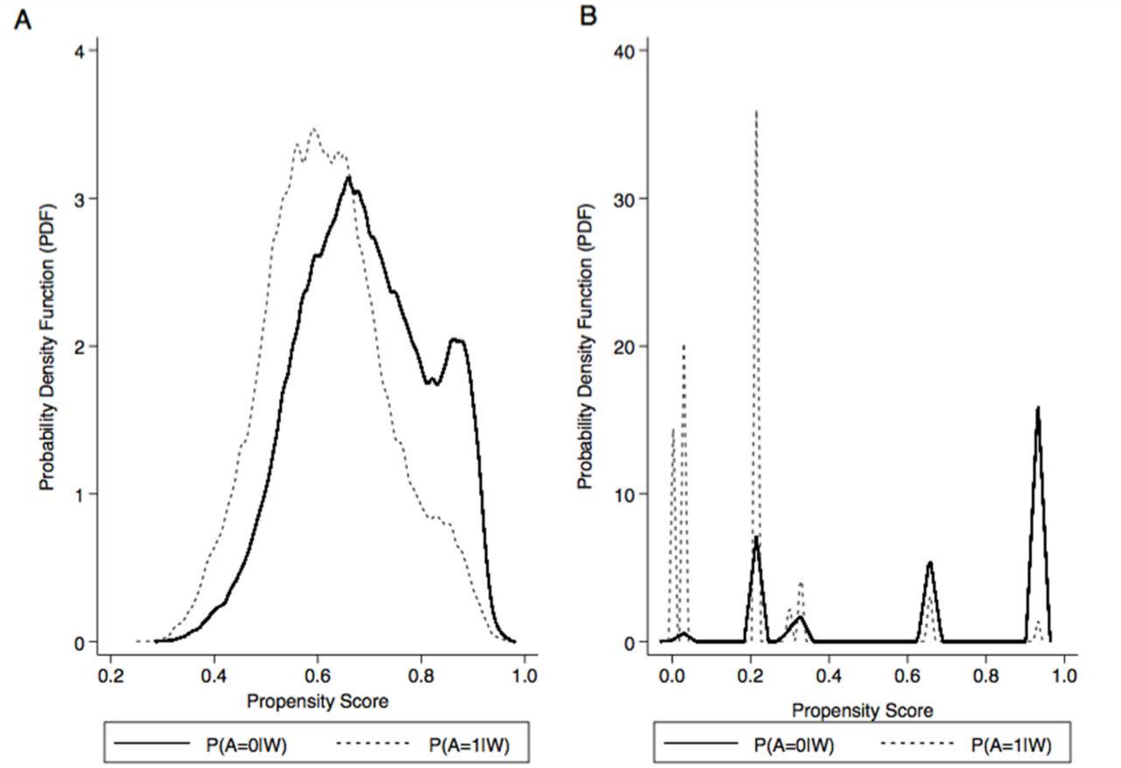

Supplement: Web Material [file kwx317luquefernandezwebmaterialfinal.pdf]
